# Recent advances in treatment of heart failure

**DOI:** 10.12688/f1000research.7022.1

**Published:** 2015-12-18

**Authors:** Takeshi Kitai, WH Wilson Tang

**Affiliations:** 1 Department of Cardiovascular Medicine, Heart and Vascular Institute, Cleveland Clinic, Cleveland, OH, USA; 2 Center for Clinical Genomics, Cleveland Clinic, Cleveland, OH, USA

**Keywords:** Heart failure, clinical trials, angiotensin-converting enzyme

## Abstract

With the total cases and economic burden of heart failure continuing to rise, there is an overwhelming need for novel therapies. Several drugs for heart failure have succeeded in preclinical and early-phase clinical trials, but most of them failed to show the real benefit in pivotal clinical trials. Meanwhile, the US Food and Drug Administration recently approved two promising new drugs to treat heart failure: ivabradine and sacubitril/valsartan. Furthermore, some of the newer agents in testing offer the potential for significant progress in addition to these drugs. Patiromer and zirconium cyclosilicate are attractive agents that are expected to prevent hyperkalemia during renin-angiotensin-aldosterone system inhibition, and serelaxin and urodilatin are promising drugs in the treatment of acute heart failure. Future clinical trials with more appropriate study designs, optimal clinical endpoints, and proper patient selection are mandatory to assess the true efficacy of these attractive compounds in clinical practice.

## Introduction and context

Heart failure (HF) is a major public health concern that affects as many as 23 million people worldwide ^1^. Furthermore, hospitalization rate and costs of care for HF are enormous, and recent years have provided few indications of improvement in these trends ^2^. There has been substantial progress in the management of chronic HF with the availability of drugs such as angiotensin-converting enzyme (ACE) inhibitors, angiotensin receptor blockers (ARBs), beta-blockers, and mineralocorticoid receptor antagonist (MRA). However, community-based outcomes for patients with HF remain suboptimal. One ongoing challenge is to ensure that proven HF therapies are used at tolerated target doses in appropriate patient populations. Because of high morbidity and mortality, there is an overwhelming need for new therapies that are safe and that can improve outcomes in patients with HF.

In 2015, the US Food and Drug Administration (FDA) approved two promising new drugs to treat HF: ivabradine and sacubitril/valsartan. In addition, some of the newer agents in testing offer the potential for significant progress. In this article, we provide a brief description of novel agents in acute and chronic HF, highlighting their mechanism of action and the clinical experience, where applicable.

## LCZ696 (sacubitril/valsartan)

### Background

Currently, blockade of the renin-angiotensin-aldosterone system (RAAS) is the cornerstone of treatment of HF. However, the combination of RAAS blockade with inhibition of neprilysin, an enzyme that degrades natriuretic peptides (NPs), has recently emerged as a potentially superior treatment strategy ^3^. In July 2015, the FDA approved sacubitril/valsartan (previously known as LCZ696) for use in patients who have chronic and stable but symptomatic HF and who have a left ventricular ejection fraction (LVEF) of less than 40%. The labeling states that the agent should be used in conjunction with other HF therapies but in place of ACE inhibitors or ARBs and is contraindicated in patients with a history of ACE inhibitor or ARB-induced angioedema.

### Mechanism of action

LCZ696 combines a neprilysin inhibitor (sacubitril) and an ARB (valsartan). Neprilysin is a zinc-dependent neutral endopeptidase that is responsible for the degradation of several vasoactive peptides such as NPs, bradykinin, and adrenomedullin and contributes to the breakdown of angiotensin II ^4^. As NPs act to promote natriuresis, diuresis, and vasodilation, neprilysin inhibition is thought to be the therapeutic target for counteracting the neurohormonal activation and complementary inhibiting the RAAS.

### Clinical efficacy

***The PARAMOUNT trial.*** The PARAMOUNT (Prospective Comparison of ARNi [angiotensin receptor-neprilysin inhibitor] with ARB on Management of Heart Failure with Preserved Ejection Fraction) trial was the first randomized controlled trial (RCT) that compared LCZ696 with valsartan in patients (n = 301) that have HF with preserved ejection fraction (HFpEF) ^5^. There was a significant decrease in NT-proBNP (N-terminal of the prohormone brain natriuretic peptide) levels in the LCZ696 group at 12 weeks; however, the difference was no longer significant at 36 weeks. Furthermore, there was no change in LV size, function, or mass; diastolic function; New York Heart Association (NYHA) class; or quality-of-life scores at 12 weeks ^5^. The trial was not designed or powered to detect clinical outcomes but has provided the rationale for the larger ongoing PARAGON-HF (Efficacy and Safety of LCZ696 Compared to Valsartan, on Morbidity and Mortality in Heart Failure Patients With Preserved Ejection Fraction) trial (ClinicalTrials.gov identifier: NCT01920711), examining the long-term outcome of LCZ696 compared with valsartan in patients with HFpEF.

***The PARADIGM trial.*** The PARADIGM-HF (Prospective Comparison of ARNi with ACE Inhibitor to Determine Impact on Global Mortality and Morbidity in Heart Failure) Trial was conducted in 8,399 patients who had NYHA class II–IV HF and an LVEF of not more than 40% and who were randomly assigned to LCZ696 or enalapril ^3^. The trial was stopped early because of an overwhelming benefit with LCZ696 therapy. The composite primary endpoint, including cardiovascular mortality and hospitalization for HF, occurred significantly more often in patients receiving LCZ696 compared with those receiving enalapril (hazard ratio 0.80, 95% confidence interval [CI] 0.73–0.87, *P* <0.001). LCZ696 was also associated with significant reductions in all-cause mortality, cardiovascular mortality, and hospitalization for worsening HF. Furthermore, those patients who received LCZ696 had lower levels of the biomarkers NT-proBNP and troponin compared with those receiving enalapril. These differences were apparent within 4 weeks of treatment and were maintained when patients were assessed again 8 months later. Interestingly, levels of B-type natriuretic peptide (BNP) actually increased and this is consistent with the mechanisms of action of neprilysin inhibition ^6^. This trial provided strong evidence for superiority of the ARNi in patients with HF with reduced ejection fraction (HFrEF) ^3^.

## Mineralocorticoid receptor antagonist

### Background

In the activity of RAAS, aldosterone is one of the most important neurohormones in the pathophysiology of HF affecting salt and water retention, endothelial dysfunction, ventricular hypertrophy, and myocardial fibrosis ^7^. Based on the results of RALES (Randomized Aldactone Evaluation Study) ^8^ and EPHESUS (Epleronone Post-Acute Myocardial Infarction Heart Failure Efficacy and Survival Study) ^9^, the guidelines recommended that the addition of low-dose MRA to optimal therapy be considered in all patients with moderate to severe chronic HF in the absence of hyperkalemia or significant renal dysfunction or both ^10, 11^. Therefore, inhibition of RAAS by MRAs, such as spironolactone and eplerenone, has become a milestone in the current HF treatment in symptomatic (NYHA class III and IV) patients with HFrEF in addition to ACE inhibitors or ARBs.

### Clinical efficacy

***The EMPHASIS trial.*** The EMPHASIS-HF (Eplerenone in Mild Patients Hospitalization and Survival Study in Heart Failure) ^12^ was a randomized, placebo-controlled study that enrolled 2,737 patients with NYHA class II with decreased LVEF under optimal recommended therapy. Patients with serum potassium of more than 5.0 mEq/l were excluded. In this study, eplerenone reduced significantly (by 37%) the primary composite outcome of risk of death from cardiovascular causes and first hospitalization for HF in comparison with placebo. The most frequent adverse event in patients receiving eplerenone was hyperkalemia.

***The TOPCAT trial.*** Thus, MRAs are highly efficacious in patients with HFrEF ^8, 12^. However, the management of HFpEF represents an ongoing challenge because therapies of proven benefit in HFrEF have repeatedly been shown to add little benefit in HFpEF ^13– 17^. The TOPCAT (Treatment of Preserved Cardiac Function Heart Failure with an Aldosterone Antagonist) trial was designed to test the clinical benefit of spironolactone in patients with HFpEF ^18^. In all, 3,445 patients with HFpEF were randomly assigned to receive spironolactone or placebo. In this trial, spironolactone failed to reduce the primary composite outcome of death from cardiovascular causes, aborted cardiac arrest, or hospitalization for HF compared with placebo (hazard ratio 0.89, 95% CI 0.77 to 1.04, *P* = 0.14). However, it did reduce the rate of HF hospitalizations (hazard ratio 0.83, 95% CI 0.69 to 0.99, *P* = 0.042) ^19^. Of interest, those enrolled in the Americas have higher event rates and followed the NP entry criteria more closely than those from Russia/Georgia and seemed to have more consistent benefits ^20^. Meanwhile, hyperkalemia was again more common in patients receiving spironolactone versus placebo (18.7% versus 9.1%, *P* <0.001). Thus, further research with a more efficient protocol is warranted to assess the efficacy of this agent in patients with HFpEF. In addition, adequate monitoring for potential side effects (mainly hyperkalemia and worsening of renal function) is needed in the addition of eplerenone to standard therapy as the current guidelines stated.

## Patiromer and zirconium cyclosilicate

### Background

As the use of RAAS inhibitors and MRAs in patients with HF increases, hyperkalemia has become a more common electrolyte disturbance in clinical practice, especially in patients with chronic kidney disease (CKD). Moreover, hyperkalemia is a major limiting factor to fully titrate these drugs in these patients who are most likely to benefit from treatment. In fact, recent clinical trials that tested the efficacy of intensive RAAS blockade had to be stopped prematurely or showed unexpected outcomes. One of the frequent adverse events was hyperkalemia ^21– 23^. Currently, non-invasive treatment of hyperkalemia is limited by a lack of safety, efficacy, and tolerability. Thus, agents to control reliably the plasma concentration of potassium while maintaining the use of RAAS inhibitors or MRAs are needed. Now, there are two novel potassium absorbents, patiromer calcium and zirconium silicate (ZS-9), that are designed to increase potassium loss via the gastrointestinal tract. Although they have not yet been approved by the FDA, both have demonstrated efficacy and safety in recent trials.

### Patiromer

***Mechanism of action.*** Patiromer is a non-absorbable polymer that binds potassium in exchange for calcium throughout the gastrointestinal tract. This agent, which is an orally administered drug, increases fecal excretion of potassium and consequently decreases plasma potassium levels ^24^. Prior patiromer clinical trials have also demonstrated the drug’s utility in treating hyperkalemia in at-risk populations for periods ranging from a few days to up to 12 weeks ^24^.

***Clinical trials.*** **The PEARL-HF study**: The PEARL-HF study tested the combined use of patiromer with spironolactone in 105 HF patients receiving standard care but with previous documented hyperkalemia or CKD. Patiromer significantly lowered serum potassium levels from baseline relative to placebo and prevented the development of hyperkalemia for more than 4 weeks in normokalemic patients with HF ^25^.

**The OPAL-HK trial**: The OPAL-HK (A Two-Part, Single-Blind, Phase 3 Study Evaluating the Efficacy and Safety of Patiromer for the Treatment of Hyperkalemia) assessed the efficacy and safety of patiromer in 243 patients with CKD on RAAS inhibitors with high levels of serum potassium. In this study, a mean reduction in plasma potassium levels was 1.0 mEq/l after the initial 4 weeks of active treatment. When patiromer treatment was stopped at the end of the active treatment period, hyperkalemia rapidly recurred over 8 weeks. The recurrence of hyperkalemia during this period was significantly higher in the placebo group than in the patiromer group (60% versus 15%, *P* <0.001), indicating the need for persistent treatment to maintain normokalemia ^24^. The most common adverse effect of patiromer therapy was constipation.

### ZS-9

***Mechanism of action.*** ZS-9 is a high-specificity inorganic crystal that entraps potassium in the intestinal tract ^26^. Instead of exchanging calcium, ZS-9 exchanges sodium and hydrogen ions for potassium. Dose-dependent excretion of potassium occurs in the feces, whereas urinary excretion decreased with dose ^27^.

***Clinical trials.*** The efficacy of ZS-9 was assessed in a multicenter RCT including 753 patients with hyperkalemia associated with a variety of diseases, including CKD, HF, and diabetes. Patients were randomly assigned to one of four doses of ZS-9 (1.25, 2.5, 5, or 10 g) or placebo for 2 days ^28^. The reduction of serum potassium with ZS-9 started acutely, and there was a dose-dependent reduction in serum potassium from baseline to 2 days, with absolute mean reductions of 0.73 and 0.53 mEq/l in the 10- and 5-g dose groups, respectively ( *P* <0.001). Reductions in serum potassium were significantly greater with ZS-9 than placebo at all time points on study day 2. Notably, 98% of patients were normalized on the 10-g dose within 2 days. The most frequent adverse effect of ZS-9 was diarrhea ^28, 29^.

**The HARMONIZE study**: The HARMONIZE study was an RCT evaluating long-term efficacy and safety of ZS-9 in 258 patients with hyperkalemia ^29, 30^. Patients achieving normokalemia (3.5 to 5.0 mEq/l) were randomly assigned to different doses of ZS-9 (5, 10, or 15 g) or placebo for 28 days in the maintenance phase. Mean baseline potassium was 5.6 mEq/l and declined to 4.5 mEq/l after 48 hours of 10-g ZS-9 treatment in the acute phase. Significant reduction in potassium was observed within 1 hour of ZS-9 administration, and 84% of patients achieved normokalemia at 24 hours and 98% at 48 hours ^30^. Furthermore, studies assessing the long-term efficacy and safety profile of this novel drug are ongoing (ClinicalTrials.gov identifier: NCT02163499).

These recent trials of patiromer and ZS-9 represented short-term safety and efficacy of these attractive therapeutic strategies in patients who develop hyperkalemia during RAAS inhibition. However, the durability of the beneficial effects and the long-term safety of these agents still have to be elucidated. In addition, there are no prospective data answering whether intensive RAAS inhibition with the use of patiromer or ZS-9 would improve the efficacy of RAAS inhibition and cardiovascular outcomes. Further study is needed to address these issues.

## Ivabradine

### Background

One novel potential therapeutic option for HF is heart rate (HR) control. An elevated HR, probably reflecting activation of the sympathetic nervous system, is associated with worse cardiovascular outcomes. Although beta-blockers are used mainly for reducing HR in HF treatment ^31^, up-titration of beta-blockers can be associated with an increased risk of adverse reactions ^32– 34^. Ivabradine, which acts by directly and selectively inhibiting the I *f* current in the sino-atrial node, has potential benefits of pharmacologic modification of HR in HF.

### Mechanisms of action

Ivabradine ****lowers HR by inhibiting a specific sinus node pacemaker I *f* current without affecting the myocardial contractility or relaxation, ventricular repolarization, or intracardiac conduction ^35– 40^. This is rather different from the mechanism induced by beta-blockers, which acts wherever beta-adrenergic receptors are present, causing negative inotropism and vasoconstriction in the bronchi; and calcium channel blockers act on the calcium channels of the heart and smooth muscle, causing negative inotropism, hypotension, and constipation.

### Clinical trials

***The BEAUTIFUL trial.*** The BEAUTIFUL (Morbidity-Mortality Evaluation of the I *f* inhibitor Ivabradine in Patients with Coronary Artery Disease and Left Ventricular Systolic Dysfunction) trial was an RCT to test the efficacy of ivabradine in 10,917 patients with stable coronary disease and an LVEF of less than 40% and an HR of more than 60 beats per minute (bpm) ^41^. In this trial, ivabradine reduced HR but had no effect on the primary endpoint of cardiovascular death or admission to a hospital for new-onset or worsening HF. However, in a subgroup of patients with an HR of at least 70 bpm, ivabradine revealed a clear benefit with respect to the secondary endpoints of admission to a hospital for a fatal or non-fatal myocardial infarction and coronary revascularization ^41^.

***The SHIFT trial.*** The SHIFT (Systolic HF Treatment with I *f* Inhibitor Ivabradine) trial was an RCT in 6,558 patients with stable symptomatic HF and an LVEF of not more than 35% in sinus rhythm with an HR of at least 70 bpm ^42, 43^. In this trial, ivabradine significantly reduced the primary endpoint of a composite of cardiovascular death or hospital admission for worsening HF and deaths due to HF ^43^. The effect was consistent across all pre-specified subgroups, including the elderly ^43^. Further analyses proved that high HR as a risk factor in HF and lowering HR improves outcomes ^43^. Other analyses showed that ivabradine reduces the risk of rehospitalization for HF ^44^ and is associated with an improvement of quality of life ^45^. HR targeted below a threshold rather than HR reduction itself has demonstrated potential benefits. One problem with interpreting the results of the SHIFT trial is that many patients were not on target doses of beta-blockers. If indeed these patients were intolerant of higher doses of beta-blockers, then these results are quite important for clinical care. Given its promising therapeutic value, ivabradine is clearly desirable in patients with symptomatic LV systolic dysfunction, elevated HR, and intolerance to beta-blockers.

## Relaxin

### Background

Serelaxin is a recombinant form of the human hormone relaxin, which is a naturally occurring hormone that is produced by the corpus luteum and placenta in pregnancy ^46^. Recent studies have shown that relaxin is also produced by the vasculature and failing myocardium ^47, 48^.

### Mechanism of action

Relaxin interacts with a G protein-coupled receptor, leading to increased cyclic adenosine monophosphate (cAMP). As a result, nitric oxide production is increased by the increased activity of inducible nitric oxide synthase (iNOS) and endothelial nitric oxide synthase (eNOS) expression ^46, 49, 50^. Additionally, relaxin upregulates the activity of vascular matrix metalloproteinase-2 (MMP-2), which can activate endothelin-1 ^51^, leading to endothelin-B receptor activation and subsequent nitric oxide production ^49^. Activation of the endothelin-B receptor is likely involved in the relaxin-mediated increases in renal blood flow ^51^. Thus, relaxin increases cardiac output, arterial compliance, and renal blood flow, supporting important physiological changes during pregnancy ^52^. Given its potent vasodilator properties as well as its ability to increase renal perfusion, relaxin became of interest as a potential therapy for acute HF.

### Clinical efficacy

***The Pre-RELAX-AHF study.*** The Pre-RELAX-AHF (Relaxin in Acute Heart Failure) study evaluated the effects of relaxin in 234 patients with acute decompensated heart failure (ADHF) within 16 hours from presentation ^53^. Patients were randomly assigned to receive four doses of relaxin or placebo for 48 hours. The key findings were that dyspnea relief and safety were optimal at 30 μg/kg per day and sustained results for dyspnea improvement. This dose also led to a substantial reduction in the composite endpoint of cardiovascular mortality or readmission due to HF or renal failure as well as a decrease in cardiovascular mortality at 180 days. However, several subjects (14%) had to discontinue relaxin therapy because of the significant fall in blood pressure ^53^.

***The RELAX-AHF study.*** The RELAX-AHF was an RCT enrolling 1,161 ADHF patients who have a systolic blood pressure of more than 125 mmHg and renal dysfunction. Patients were randomly assigned to receive serelaxin 30 μg/kg per day or placebo as a continuous 48-hour infusion within 16 hours from presentation ^54^. In this study, serelaxin significantly improved dyspnea, shortened the length of hospital stay, and decreased the incidence of worsening HF as compared with placebo. There was also an improvement in the 6-month mortality outcomes and no evidence of adverse effects of this agent on kidney function ^55^. Although relaxin has shown success in improving the clinical course of patients with ADHF during the initial hospitalization with an acceptable safety profile, a larger trial (RELAX-AHF2, n = 2,685) is ongoing to hopefully validate whether this drug could indeed provide long-term mortality benefit.

## Ularitide

### Background

Decongestion is an important part of managing both acute and chronic HF, and retention of fluid and sodium metabolism play a fundamental role in this. NPs are activated in HF and exert compensatory effects by inhibiting the RAAS and inducing vasodilation and natriuresis ^56^. Therefore, NPs have received much interest as a potential therapy in ADHF. NPs consist of atrial NP (ANP), BNP, C-type NP (CNP), D-type NP (DNP), and urodilatin ^57^.

### Mechanism of action

Urodilatin was first isolated from human urine in 1988 as a modified version of pro-ANP ^58^. It is produced mainly by distal renal tubule cells and is secreted into urine and is involved in renal sodium handling ^59^. Synthetic NPs such as carperitide (a recombinant form of ANP) and nesiritide (a recombinant form of BNP) are currently used to treat congestive HF (carperitide is available only in Japan). When it is administered to patients with ADHF, a rapid reduction of pulmonary capillary pressure and consequent relief of dyspnea often result because of natriuresis, diuresis, and venous and arterial dilation. However, NP-induced vasodilatation and reductions in renal perfusion pressures and the potential for reflex sympathetic responses can cause clinically significant systemic hypotension and worsening of renal function in some patients ^60^. In contrast to ANP and BNP, urodilatin is effective in more distal parts of the renal tubular system because of its slower elimination rate ^61^.

### Clinical efficacy

***The SIRIUS II study.*** The Prospective Double-blind Study in Patients with Symptomatic, Decompensated Chronic Heart Failure (SIRIUS) II study was aimed to assess the clinical effects of ularitide in 221 patients with ADHF ^59^. The primary endpoint was a significant decrease in pulmonary capillary wedge pressure (PCWP) as well as improvement in dyspnea at 6 hours after completion of the 24-hour infusion. Ularitide demonstrated a significant reduction of PCWP for all three dosage groups (7.5, 15, and 30 ng/kg per min). At higher doses, the agent reduced systemic vascular resistance and increased cardiac index. Besides the beneficial hemodynamic effects, improvement in dyspnea was reported. The most frequently reported drug-related adverse events were dose-dependent blood pressure decrease. Currently, a randomized, placebo-controlled, phase 3 study—Efficacy and Safety of Ularitide for the Treatment of Acute Decompensated Heart Failure (TRUE-AHF), n = 2,152—is ongoing to measure the effect of 48-hour infusion of ularitide.

NPs offer us a unique and attractive strategy for HF treatment, acting as diuretic, natriuretic, vasoactive agents without any inotropic or chronotropic effects. However, the future role of NPs in ADHF therapy is still not yet clear, especially following the rise and fall of nesiritide use. Initial trials with ularitide, a synthetically produced urodilatin, showed hemodynamic and clinical benefits in patients with ADHF. Although ularitide has potential to be an alternative to nesiritide or carperitide, much more evidence is needed to evaluate the role of this agent in HF therapy.

## Conclusions and future perspectives

A number of promising compounds for HF therapies are under investigation in addition to the agents we discussed here. However, it is a well-known fact that several drugs have succeeded in preclinical and early-phase clinical trials only to be disappointments in pivotal clinical trials. Therefore, future clinical trials with adequately powered, more appropriate study designs, optimal clinical endpoints, and right patient selection are mandatory to assess the true efficacy of these compounds.

## Abbreviations

ACE, angiotensin-converting enzyme; ADHF, acute decompensated heart failure; ANP, atrial natriuretic peptide; ARB, angiotensin receptor blocker; ARNi, angiotensin receptor-neprilysin inhibitor; BNP, brain natriuretic peptide; bpm, beats per minute; cAMP, cyclic adenosine monophosphate; CI, confidence interval; CKD, chronic kidney disease; FDA, US Food and Drug Administration; HF, heart failure; HFpEF, heart failure with preserved ejection fraction; HFrEF, heart failure with reduced ejection fraction; HR, heart rate; LV, left ventricular; LVEF, left ventricular ejection fraction; MRA, mineralocorticoid receptor antagonist; NP, natriuretic peptide; NT-proBNP, N-terminal of the prohormone brain natriuretic peptide; NYHA, New York Heart Association; PCWP, pulmonary capillary wedge pressure; RAAS, renin-angiotensin-aldosterone system; RELAX-AHF, Relaxin in Acute Heart Failure; RCT, randomized controlled trial; SHIFT, systolic heart failure treatment with I *f* inhibitor ivabradine.
